# DNA Methylation in Plant Responses and Adaption to Abiotic Stresses

**DOI:** 10.3390/ijms23136910

**Published:** 2022-06-21

**Authors:** Minghui Sun, Zhuo Yang, Li Liu, Liu Duan

**Affiliations:** State Key Laboratory of Biocatalysis and Enzyme Engineering, Hubei Collaborative Innovation Center for Green Transformation of Bio-Resources, Hubei Key Laboratory of Industrial Biotechnology, School of Life Sciences, Hubei University, Wuhan 430062, China; minghuisun@stu.hubu.edu.cn (M.S.); yangzhuo@stu.hubu.edu.cn (Z.Y.)

**Keywords:** plant epigenetics, DNA methylation, abiotic stresses, stress memory

## Abstract

Due to their sessile state, plants are inevitably affected by and respond to the external environment. So far, plants have developed multiple adaptation and regulation strategies to abiotic stresses. One such system is epigenetic regulation, among which DNA methylation is one of the earliest and most studied regulatory mechanisms, which can regulate genome functioning and induce plant resistance and adaption to abiotic stresses. In this review, we outline the most recent findings on plant DNA methylation responses to drought, high temperature, cold, salt, and heavy metal stresses. In addition, we discuss stress memory regulated by DNA methylation, both in a transient way and the long-term memory that could pass to next generations. To sum up, the present review furnishes an updated account of DNA methylation in plant responses and adaptations to abiotic stresses.

## 1. Introduction

Most plants are in a sessile state during the entire growth cycle, and they cannot avoid the stress of the natural environment as sensitively as animals. In order to better adapt to the environment and improve the survival probability, plants have formed a series of complex mechanisms. Among them, the regulation of plant responses to external stresses by epigenetic mechanisms is one of the important mechanisms discovered in recent years. Abiotic stress could induce epigenetic changes at many different levels, transmit stress signals, and regulate plants stress responses [1]. Epigenetic changes involve three main mechanisms: DNA methylation, histone modifications, and RNA-mediated gene silencing [2]. DNA methylation is one of the earliest discovered and most studied regulatory mechanisms in epigenetics, and is considered to be a relatively stable, heritable, transgenerational mark, involving a series of biological processes such as temporal and spatial gene expression, transposable element activity, and genomic imprinting [3].

DNA methylation generally refers to the transfer of a methyl group onto the C5 position of the cytosine to form 5 methylcytosine (5mC) [4]. In mammals, the main type of DNA methylation is CG methylation, while non-CG methylation is limited in specific tissues such as in embryonic stem cells [5,6]. In contrast, both CG and non-CG methylation, such as DNA methylation in CHG (symmetric) and CHH (asymmetric) environments (H = A, C, or T), are ubiquitously detected in higher plants [3]. DNA methylation is not limited to the promoter regions of genes, but also their coding regions. There are three processes of DNA methylation involved in plants: DNA methylation maintenance (the methylation of hemimethylated symmetrical sequences), de novo DNA methylation (methylate at a previously unmethylated C), and DNA demethylation (the methylation state can be reversed). The methylome in plants is primarily maintained during DNA replication and cell division by DNA methyltransferases, including maintenance methylases and de novo methylases [7]. The maintenance of DNA methylation of symmetrical sites, such as CG and CHG sequences, is completed through the methyltransferase 1 (MET1) [8], and chromomethylase 3 (CMT3) [9], respectively. The RNA-directed DNA methylation (RdDM) pathway and chromomethylase 2 (CMT2) are necessary for maintaining CHH methylation. The methylation of asymmetric site CHH is achieved by de novo methylases, via domains rearranged methyltransferase 1 (DRM1) and domains rearranged methyltransferase 2 (DRM2) by small RNAs through RdDM [10]. The RdDM pathway and the action of RNA interference (RNAi) machinery, DRMs, CMT3, and RNA polymerases are required in de novo methylation processes of all sequence contexts. DNA demethylation can be divided into active and passive processes: passive DNA demethylation is caused by the reduction or inactivation of enzymes important in DNA methylation during DNA replication, while the mechanism of active DNA demethylation is a complex process mediated by the base excision repair (BER) pathway. Active DNA demethylation in plants involves 5mC DNA glycosylase, and studies in *Arabidopsis* have shown that repressor of silencing 1 (AtROS1), DNA glycosylases demeter (AtDME), and demeter-like proteins 2 and 3 (DML2 and DML3) take roles in DNA demethylation [11,12,13].

Several methods could be used to detect DNA methylation, including methods based on antibodies or specific proteins with an affinity for methylcytosine, such as methylated DNA immunoprecipitation (MEDIP) and the capture of methylated DNA by methyl-CpG binding domain-based (MBD) proteins (MBDCap) [14], methods using methyl-sensitive restriction enzymes, such as methyl-sensitive amplification polymorphism (MSAP) [15,16], and methods involving the treatment of DNA with sodium bisulfite [17], such as whole-genome bisulfite sequencing (WGBS). According to their resolution and cost, affinity enrichment-based methods are suitable for rapid, large scale, and low-resolution studies, restriction enzymes-based methods are suitable for site-specific/targeted studies, and bisulfite conversion-based methods are suitable for high resolution studies.

Abiotic stresses include high temperature stress, cold stress, drought stress, salt stress, and heavy metal stress, which threaten plant growth and result in reduced crop yields and species diversity. Existing studies have shown that DNA methylation is a mechanism by which plants adapt to abiotic stresses [18,19]. Different stresses can trigger specific and dynamic DNA methylation changes in plants, regulating gene expression levels of stress-responsive genes, and controlling the activity of transposable elements (TEs), thereby inducing and improving the response and adaption of plants to stresses [20]. Most abiotic stress-induced DNA methylation modifications are transient and return to initial levels upon stress elimination; however, studies have also shown that some short-term or long-term “memory effect” could be induced in plants [21]. Due to transient changes in morphological and biochemical metabolites, short-term stress memory allows plants to maintain stress resistance for a short period of time or throughout their life, whereas long-term stress memory may be transferred to offspring. DNA methylation causes heritable epigenetic modifications in the absence of sequence changes, and the methylation changes in the offspring of stressed plants related to the parental environment [21].

This review summarizes the different roles of DNA methylation in plant responses to abiotic stresses and proposes the future development direction of DNA methylation research, which facilitates the development of stress-resistant plants that can deal with abiotic stresses such as drought, high temperature, cold, salt, and heavy metals.

## 2. DNA Methylation and Drought Stress

Drought is the most damaging environmental stress on plant yield and growth rate in the past few decades, due to climate change [22,23]. Plants have evolved complex mechanisms in response to drought stress, and DNA methylation regulation plays a pivotal role in regulating gene expression. By studying the methylation status of a single cytosine in the whole genome under drought stress, the overall methylation level of plants under drought stress are higher than that of control plants, such as 8.64% higher in mulberry (*Morus alba*) and 2.29% higher in *Populus trichocarpa* [24,25].

Correlation analysis has shown that DNA methylation has multiple effects on gene expression under drought stress, indicating that it directly or indirectly affects gene expression through multiple regulatory pathways. Drought-tolerant plants were found to have a more stable methylome under drought conditions, with differentially methylated regions (DMRs)-related genes mainly related to the stress response, programmed cell death, and other pathways in rice (*Oryza sativa* L.) [26], mulberry (*Morus alba* L.) [27], mungbean (*Vigna radiata* L.) [28], and maize (*Zea mays* L.) [29]. For example, an in-silico genome-wide DNA methylation (5mC) analysis was performed in rice (*Oryza sativa* cv. *Zhonghua11*) where 14 unique genes of the eukaryotic gene superfamily cytochrome P450 with different methylation levels were identified in the rice genome under drought stress [30], cytosine methylation at a single-base resolution and methylation patterns associated with water scarcity in representative drought-sensitive and drought-tolerant varieties in the genome of commercial apple (*Malus x domestica*) [31], and so on.

Global methylation and transcription analysis revealed that promoter unmethylated genes were expressed at higher levels than promoter methylated genes. In maize, DNA methylation in the *ZmNAC111* promoter represses *ZmNAC111* expression, resulting in an increased drought sensitivity [32]. Drought stress is also associated with changes in the methylation of the gene body of many genes, including those encoding transcription factors (TFs). A negative correlation between gene body methylation and gene expression was found in maize roots under water stress [29]. Changes of DNA methylation occurs in TEs as well. In poplar (*Populus trichocarpa*), transcription factors affecting gene expression after drought treatment were affected by methylated transposons. Methylated transposons involved in drought signal transduction pathways were found in C2C2, WRKY, MYB, and other families [25].

Cytosine-5-methyltransferases and demethylases are two important enzymes that play an important role in dynamically maintaining the DNA methylation status of plant genomes under drought stress. Drought stress induced the up-regulation of 5mC methyltransferase and demethylase in plants, such as apple (*Malus x domestica*) [31], tomato (*Solanum lycopersicum*) [33], and eggplant (*Solanum melongena* L.) [34].

The degree, level, and polymorphism of plant DNA methylation under water-deficient conditions were found to exhibit tissue-specific and genotype-specific characteristics. Higher levels of DNA methylation and demethylation, and higher methylation polymorphisms were found in wheat (*Triticum aestivum* L.) drought tolerant genotype AK58 compared with those of the common wheat genotype MinMai 13 [35]. In addition, methylation polymorphisms in the root were higher than that in the leaf under a water deficit, especially in AK58, which might be one of possible explanations that AK58 responds more quickly to water deprivation through changes in DNA methylation [35].

### 2.1. DNA Methylation and High Temperature Stress

High temperature stress is a serious threat to crop growth and development worldwide, which causes a series of morphological, physiological, and biochemical changes in plants. As a result, nearly all organisms have evolved signaling pathways to sense changes in ambient temperature and adjust their metabolism and cellular functions to prevent heat-related damage [36]. Recently, great progress has been made in the epigenetic regulation of thermal responses, including DNA methylation [37,38].

Results have shown that the overall methylation level of plants under heat are lower than that of control plants in most cases, such as in *Populus simonii* and *Brassica napus* [39,40]. Cytosine methylation changes in a large number of different genes were affected by heat stress. Under heat treatment, the degree of methylation of heat-sensitive genotypes was higher than that of heat-resistant genotypes, and more DNA demethylation events occurred in heat-resistant genotypes, while more DNA methylation occurred in heat-sensitive genotypes [41]. Studies have shown that heat stress induces DNA demethylation in genes in *Arabidopsis thaliana*, rather than in the intergenic regions [42]. There is also strong evidence that TEs, which have been implicated in the up-regulation of gene expressions under heat stress [43], can be induced or activated in response to heat stress [44,45]. For example, the LTR-copia-type retrotransposon *ONSEN* in *Arabidopsis* is activated by heat stress [46]. DMRs were identified in both gene sequences and promoter regions under both mild heat stress and severe heat stress, as well as in mitochondrial DNA in Arabidopsis [47].

DNA methylases and demethylase genes play critical roles in dynamically maintaining the DNA methylation status of plant genomes under heat stress. Twenty-two DNA methylase genes and six DNA demethylase genes were identified in the rapeseed (*Brassica napus* L.) genome [48]. Expression analysis by RNA-seq and qRT-PCR indicated that these DNA methylation/demethylation-related genes may be involved in the heat/salt stress response of rapeseed [48]. Xia Shen et al., studied two *Arabidopsis* germplasms from Eurasia and discovered 16 novel loci, including an association between CMT2 and temperature seasonality. *cmt2* deletion mutants showed a higher tolerance to heat stress, strongly suggesting a role for the genetic regulation of epigenetic modifications in natural adaptation to temperature [49]. Notably, DNA (de)methylation may be a key regulatory process to ensure the proper germination of seeds produced under heat stress. In *Arabidopsis*, gene expression is strongly altered under severe heat stress during seed development, promoting heat stress response mechanisms. It was observed that DNA demethylation caused by the *ROS1* gene could impair seed germination by affecting the expression of germination-related genes. On the other hand, under severe heat stress, most of the DMRs are located in the promoters and gene sequences of germination-related genes [47].

### 2.2. DNA Methylation and Cold Stress

Freezing or extremely low temperatures are key factors affecting plant growth, development, and crop yield. In response to cold stress, plants develop several mechanisms to minimize the potential damage caused by low temperature [50]. DNA methylation changes are an important way for plants to regulate gene expression in response to cold stress [51,52].

Cold exposure resulted in significantly lower DNA methylation levels in sugar beet [53]. Total methylation was decreased under high chill conditions, while no significant decrease was found in low chill conditions in apple (*Malus x domestica Borkh*.) [54]. Under cold stress, the tolerant genotype prevented the accumulation of H_2_O_2_, resulting in lower damage indices, such as malondialdehyde and electrolyte leakage indices, compared with the sensitive genotype [55]. For prolonged cold stress, the changes of demethylation bands in tolerant genotypes were higher than those in sensitive genotypes, indicating a higher activation potential of cold-stress-responsive genes in tolerant genotypes in chickpea [55].

As a direct and/or indirect product of gene expression regulated by different factors such as DNA methylation, cold stress signals are translated into physiological changes. Genes involved in cellular metabolism, the stress response, the antioxidant system, the lysine metabolic pathway, and transcriptional regulation showed a correlation between methylation and their expression in chickpeas and tartary buckwheat [55,56]. The decrease in DNA methylation was accompanied by the transcriptional down-regulation of the *CMT2* gene and strong up-regulation of several genes mediating active DNA demethylation such as *HbICE1*, *HbCBF2*, and *HbMET* [53].

Both methylation and demethylation occur during cold adaptation. In *Brassica*, totally 1562 differentially methylated genes were identified during cold acclimation, including *BrammDH1*, *BraKAT2*, *BraSHM4,* and *Bra4CL2*, whose promoters were demethylated and resulted in an increase in their transcriptional activity [57]. It was found that in the rice cold-tolerant variety P427, 51 genes showed both methylation and expression level changes under cold stress, involved in the ICE–CBF–COR (CBF expression inducer—C-repeat binding factor—cold regulation) pathway and plays a crucial role in cold tolerance [58]. It was found that cold stress may lead to decreased DNA methylation in the promoter of the homologous gene of the open stomatal 1 in rice (*Os03g0610900*), which could interact with and phosphorylates ICE1, and increases its gene expression [58]. The correlation between gene body methylation and gene expression during chilling dormancy in apple was analyzed by bisulfite sequencing and qRT-PCR. Low temperature was associated with the hypermethylation of gene bodies, which may lead to the repression of their expression [54]. It was also found that TE families may be associated with the triggering of the cold stress-responsive expression of nearby genes, with responses highly variable between genotypes [44].

DNA demethylation take roles in cold responses as well. Cold treatment increased the transcriptional activity of cold-related genes and cold-responsive genes, such as *HbICE1*, *HbCBF2*, accompanied with induced expression of DNA methylation related genes, and also induces DNA demethylation of their promoters in rubber tree (*Hevea brasiliensis*) [59], *Brassica rapa* [57], tomato [60], and sweet orange (*Citrus sinensis* L.) [61]. Cold stress also resulted in a reduction in DNA methylation levels in the CHH context, accompanied by transcriptional down-regulation of *CMT2*, and the strong up-regulation of several genes mediating DNA demethylation [53]. Reduced genomic DNA methylation in the apex tissue of poplar (*Populus* L.) was found to be correlated with the induction of chilling-dependent *DEMETER-LIKE DNA demethylase 10*, which was involved in bud break [62].

### 2.3. DNA Methylation and Salt Stress

Soil salinization has become a serious environmental problem, threatening sustainable agriculture and future food security [63]. High salinity negatively affects osmotic and ionic balance, protein synthesis, photosynthesis, energy, and lipid metabolism [64]. Accumulating evidence suggests that DNA methylation plays an important role in regulating the gene expression response to salinity [65].

Salinity induces genome-wide changes in DNA methylation status, and different effects on DNA methylation in diverse plant species or specific genes were induced by salt stress [66]. Salinity stress increased the methylome content of alfalfa (*Medicago* spp.) plants, and the treatment with 5-AzaC (a DNA methylation inhibitor) on alfalfa seedlings resulted in a significant decrease in salt tolerance [67]. On the other hand, CG methylation levels were significantly reduced in the genomic regions analyzed within the epidermis under NaCl stresses, and the reduction was more robust in severely stressed *Arabidopsis* plants [68]. An increase in 5mC has been detected in CHG and CHH in the shoot under a salt-sensitive wheat genotype, while reduced 5mC levels were found in a salinity-tolerant wheat cultivar SR3 [69]. In rice, hypermethylation was found in tolerant genotypes, whereas sensitive genotypes displayed demethylation [70]. Different levels of DNA methylation were found in the root and shoot system during salt stress in rice and wheat [71,72]. In olive (*Olea europaea* subsp. *europaea* var. *europaea*) plants, DNA methylation levels increased when plants were subjected to salt stress. These changes were more pronounced in salt-tolerant cultivars, with higher DNA methylation events in royal cultivars than in Koroneiki [73]. These findings further indicated the possible regulatory roles of DNA methylation in conditioning the tolerance to high salinity depending on different species and tissues.

Under salt stress, DNA methylation regulates the expression of genes, including membrane transporter genes, heavy metal transporter genes, and organic acid secretion genes, thereby controlling stress signals and causing stress responses in plants. A genotype- and tissue-specific increase in cytosine methylation on the high-affinity potassium transporters *TaHKT2;1* and *TaHKT2;3* under NaCl treatment, were found down-regulated in wheat genotype Kharchia-65, contributing to the improved salt-tolerance ability [74]. Methylation in salinity responsive genes were found could induce the salinity tolerance of plants, such as the flavonol synthase genes *TaFLS1* and *TaWRS15* in wheat and barley (*Hordeum vulgare*) [75].

DNA demethylation plays crucial roles in salt stress as well. The exposure of plants to salt stress induced the expression of genes encoding enzymes of the flavonoid biosynthesis pathway (CHS, CHI, F3H, FLS, DFR, ANS) and the antioxidant pathway (GST, APx, GPx, GR), which correlated with their methylated status and *AtROS1* demethylase activity [76]. Salt stress reduced the CG methylation level of the *Glabra-2* (*GL2*, a master gene associated with root epidermal cell differentiation) in its gene body region, which related to its lower expression levels [68]. Compared with IR64 in rice, the expression of *OsBZ8* (Abscisic acid Responsive Element -binding factor) was highly induced in the salt-tolerant variety Nonabokra under salinity stress, along with the loss of DNA methylation was observed at OsBZ8 locus [77].

The majority of the gene promoters exhibiting changes in methylation were hypermethylated under salt stress, and gene bodies in the progeny of stressed plants as well, accompanied with most of the hypermethylated genes having a lower gene expression in Arabidopsis [78]. Furthermore, cytosine alterations found in the UTRs and exons of rice under salinity stress indicated a significant role of gene body methylation in regulating gene expression [79].

Salt stress can affect the expression of *CMT*, *DNMTs*, *DRMs*, *DMEs*, and *DMLs* and induce methylation variation in plant DNA, providing plasticity for plants to adapt to salt stress. The expression levels of some members of the *CMT* and *MET* family were significantly down-regulated in response to salt stress, while *DNMT2* showed up-regulation in rapeseed (*Brassica napus* L.) [48]. Genes in the *DRMs* family were down-regulated in response to salt stress, while *BnaDRMa*, *BnaDRMg*, and *BnaDRMh* were significantly up-regulated after salt treatment, and most demethylase genes such as *DMEs*, *DML3s*, and *ROS1* were mildly up-regulated in rapeseed [48]. Furthermore, it was reported that in *P. betulaefolia*, the expression of *DME* responds to salt in a tissue-specific pattern, with down-regulation in leaves and up-regulation in roots [80]. Overexpressing the *Arabidopsis* demethylase gene *AtROS1* in tabacco increases the demethylation levels of both promoters and gene bodies of genes in flavonoid biosynthetic and antioxidant pathways under salt stress, which showed a higher gene expression and a higher tolerance to salt stress [78].

It is worth noting that various single salt treatments and their mixed salts may have different effects on plants. Research found that in the halophyte *Chloris Virgata*, the effects of salt on DNA methylation was ranked as Na_2_CO_3_ > NaHCO_3_ > Na_2_SO_4_ > NaCl, and the mixed salts showed tissue-specific effects. Furthermore, they concluded that mixed salts are not a simple combination of a single salt [81].

### 2.4. DNA Methylation and Heavy Metal Stress

Industrial pollution has led to changes in the balance of some heavy metals, and excessive accumulation of heavy metals in soil is toxic to most plants [82]. Plant roots absorb excess heavy metal ions from the environment and transfer them to their shoots, which affects their metabolism and hinders their growth [83]. Under heavy metal stress, there is a close relationship between physiological responses, gene expression levels, and DNA methylation patterns [84]. Much attention has been paid to decipher the mechanisms by which plants resist heavy metal stress.

**Figure 1:**
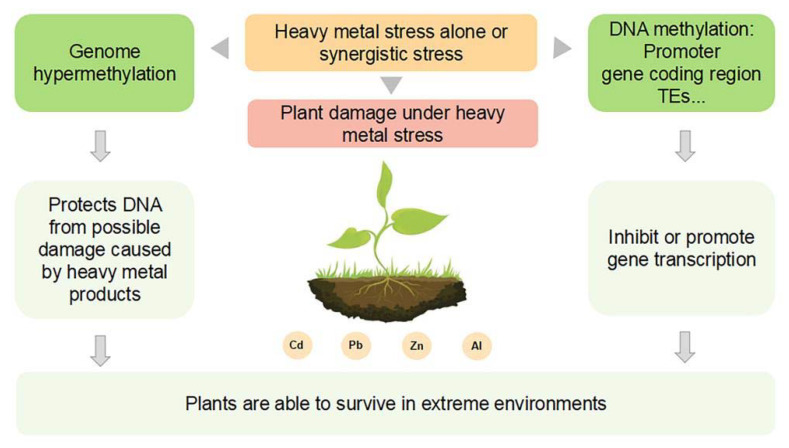
DNA methylation and heavy metal stresses. Under heavy metal stress alone or in concert, DNA methylation may play a role in regulating plant responses to heavy metals through at least two mechanisms. The first mechanism is hypermethylation, a defense strategy of plants that can prevent genomic instability and protect DNA from possible damage by heavy metal products, allowing plants to survive in extreme environments. The second mechanism involves gene expression regulation, which is not limited to the promoter regions of genes, but also their coding regions.

Hypermethylation has been viewed as one of the defense strategies for plants to protect themselves from possible damage by heavy metal products, allowing them to survive in extreme environments (Figure 1). DNA methylation changes induced by cadmium (Cd) stresses were analyzed in rice plants (*Oryza sativa* ssp *japonica* cv. *Nipponbare*), and more hypermethylated genes were found than hypomethylated ones [85]. The level of total methylation in radish and soybean (*Glycine max*) increased after Cd exposure with a significant dose-dependent relationship [86,87]. Methylation levels in the roots of heavy metal tolerant plants is significantly higher than that of sensitive plants, comparing clover (*Trifolium repens* L., sensitive species) with hemp (*Cannabis sativa* L., partial tolerant) [88], as well as Ni-tolerant *Noccaea caerulescens* with Ni-susceptible *A. thaliana* [89]. However, DNA hypomethylation was observed in the wheat resistant variety Pirsabak 2004 in response to lead (Pb), Cd, and zinc (Zn), in promoter regions of metal detoxification transporters [90]. For heavy metal such as Aluminum (Al), the exposition induced demethylation in both tolerant and non-tolerant plants, while Al stress triggered DNA hypermethylation as a protective response in *Zea mays* [91]. Furthermore, it was reported that in triticale lines, DNA methylation increased in Al tolerant lines and reduced in non-tolerant lines [92]. The complicated responsive patterns between them reveal that there might be different mechanisms for plants to protect themselves depending on whether the heavy metal element is essential for plant growth or not, or depending on the plant species and their developmental stages.

Gene expression controlled by DNA methylation is another epigenetic response to heavy metal stress. In rice after Cd treatment, most of the DNA methylation modified genes show altered transcription levels, and methylation patterns were closely associated with transcriptional differences in stress-responsive genes involved in metal transport, metabolic processes, and transcriptional regulation [85]. An analysis of maize roots under normal Pb treatment revealed that 140 genes exhibited an altered DNA methylation status, including some well-known stress-responsive transcription factors and proteins such as MYB, AP2/ERF, bZIP, serine-threonine/tyrosine-protein, pentapeptide repeat protein, RING zinc finger protein, F-box protein, leucine-rich repeat protein, and tetrapeptide repeat protein [84]. Of the 30 differentially methylated DNA fragments of characterized soybean after Cd stress, fifteen were found associated with plant stress responses [87].

Methylation on gene promoters usually inhibits gene transcription, but in some cases it can also promote it under heavy metal stress [93]. In barley, 97.8% of whole cytosine of the promoter of *HvAACT1*, a major gene responsible for exogenous detoxification of Al, was unmethylated in the Al-tolerant cultivar FM404, whereas the Al-sensitive cultivar SV239 showed a much higher rate of methylation in the promoter [94]. The regulation by DNA methylation under heavy metal stresses is not limited to the promoter regions of genes, but also their coding regions and TEs. DNA methylation and DMRs were found in upstream, gene body, and downstream regions in rice after Cd treatment [85]. In maize root under Pb stress, results of whole-genome bisulfite sequencing showed that the average methylation density of the introns was higher than the UTRs and exons [74]. Transposable elements are one of the most heavily methylated DNA regions and were shown to play a role in Al stress responses in tolerant accessions of barley [94]. Furthermore, it was also found that a high density of TEs were strongly methylated in radish under Pb treatment [86].

DNA methylation and demethylation were found in response to heavy metals. The upregulation of *CMT1* was found related with induced DNA hypermethylation in *Posidonia oceanica* after Cd treatment [95]. Mutation of *MET1* and *DRM2* resulted in significantly reduced transcript levels of the genes such as *OsIRO2* and *OsPR1b* in rice seedlings under Cd stress as well, suggesting that DNA methylation participated in the plant response to Cd [85]. Moreover, heavy metals uptake and translocation have an interplay and coordination effect and recent findings have shown that Zn, Pb, and Cd could differentially regulate the expression level of DNA methyltransferases and the DNA methylation levels in maize, individually and in combinations [96].

## 3. DNA Methylation and Stress Memory

Plants are continuously challenged by different environment stresses in their lifetime, and interestingly, some plants could become more tolerant during subsequent stresses to the similar stress after a first mild exposure to better survive. In this way, it is widely accepted that plants have “stress memory”. Therefore, processes such as priming are proposed as a promising approach and have been used for plant adaption to future exposure through the acquisition of memory in plants, such as in *Zea mays* [97], *Arabidopsis* [98], and *Brachypodium distachyon* [99].

**Figure 2:**
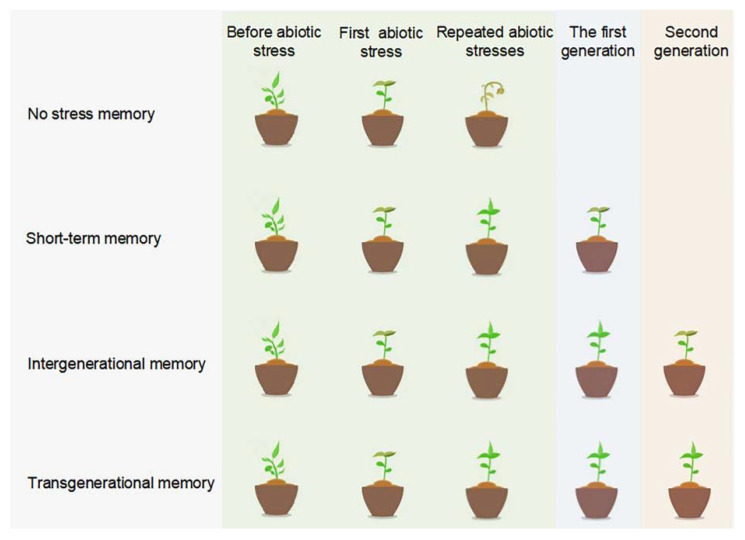
Plant memory in abiotic stresses. Plants that have not obtained stress memory in the first stress could not survive after repeated abiotic stresses. Plants that gain a short-term memory could enhance their resistance to the second stress, but only last for a short time or during the lifespan, not possible to pass on to the progeny. Plants that gain intergenerational memory exhibit stress memory only in the first stress-free offspring generation. The stress memory is heritable and observable for at least two generations in plants that achieve transgenerational memory.

Sometimes, transgenerational priming effects could be observed between generations [100], passing down the memories to offspring and are inheritable. Therefore, the stress memory induced in plants could be classified as short-term stress memory and long-term stress memory (Figure 2). Short-term stress memory allows plants maintain resistance to stress in a short time or throughout the lifespan, due to temporary changes in morphology and biochemical metabolites [101], while long-term stress memory could be transferred to offspring. Among long-term stress memory, memory only passed on to immediate offspring is called intergenerational stress memory, while some stress responses could be remembered for at least two subsequent stress-free generations, called transgenerational stress memory [102].

In recent years, epigenetic modulations such as DNA methylation have been recognized as important components in stress memory, enabling plants to respond efficiently to recurring stress and prepare their offspring for potential stresses. It has been observed that the DNA methylation level changes during exposure to stress conditions, and the effects could be repeatedly established. During the recovery stages after stress conditions, some methylation changes were gradually restored while some others remained for a better and quicker response against stresses. After cycles of mild drought and re-watering treatment in rice, short-term drought stress memory was established, and genes that participated in the rice drought short-term memory response were identified and grouped into 16 distinct memory patterns [103]. Transcriptome analysis and whole genome bisulfite sequencing results showed a linkage between DNA methylation and drought memory transcripts, which provide evidence that DNA methylation participated in plant stress short-term memory formation. Furthermore, methylation status was found highly dynamic throughout stress duration and recovery. Memory DMRs could directly regulate rice drought memory genes and were associated with drought stress short-term memory [104].

DNA methylation could also be stably inherited through generations, and play important roles in plant long-term stress memory, including intergenerational and transgenerational memory (Figure 2). An analysis of the DNA methylation patterns in leaf tissues of rice (*Oryza sativa*) plants treated with heavy metals showed that CHG demethylation status could be inherited via the maternal and paternal germline and was often accompanied by further hypomethylation [105]. Intergenerational stress memory was also found associated with DNA methylation in species other than rice, such as in *Arabidopsis* [106], oilseed rape (*Brassica napus*) [107], soybean [108], and maize under various abiotic stresses [109]. A high proportion of drought induced DNA methylation status that was maintained in advanced generations, resulted in transgenerational stress memory, and improved the drought adaptability of rice offspring in upland fields [110]. Differential methylation between maize inbred lines is heritable, and DMRs could shift from one epiallele to others stably inherited in recombinant inbred lines [111]. Weixuan Cong et al., investigated a Tos17 retrotransposon for its methylation state for three generations and found that the DNA methylation in response to heavy metal stress was transgenerational inherited [100]. The heritability of DNA methylation was also found in the annual plant *Polygonum persicaria*. Their offspring of drought-stressed parents developed longer root systems and gained greater biomass than the offspring of well-watered parents of the same genetic line [112].

However, the inheritance of long-term memory varies in how many generations it lasts. The effect of salt stress on DNA methylation of *Thlaspi arvense* was investigated at the population level under the stimulation of salinity stress, and results showed that their stress memory could pass to at least two generations of offspring in a non-stress environment [113]. Xiaoguo Zheng et al., evaluated the DNA methylation of the original generation and the sixth generation of two rice varieties with different drought resistance levels under drought stress. The results showed that DNA methylation induced by drought stress could be preserved in the subsequent six generations and the drought stress had a cumulative effect on the DNA methylation patterns of both varieties [114]. Multigenerational exposure was performed to understand epigenetic variations in offspring. Ten generations of cultivation under salt stress could increase 45% spontaneous epigenetic changes in *Arabidopsis* [115]. Over 25 consecutive generations repeatedly exposed to heat stress, *Arabidopsis* line F25H was compared with its parental and parallel control progenies, results showed significantly lower global methylation levels of CHH and CHG in F25H than in control plants and more pronounced methylation changes in the gene body than TEs in F25H stressed progeny [116]. Hence, DNA methylation induced by heat stress in the progeny could result in a phenotypic resilience to adverse environments under long-term stress conditions.

## 4. Concluding Remarks

Continuous environmental changes pose serious abiotic threats to plant survival, and certain environmental stresses are repetitive, such as heat, drought, salinity, etc. Being sessile in nature, plants have evolved a complex response to these unfavorable and recurrent environmental conditions that involve transcription control, epigenetic regulation, and physiological and metabolic reprogramming for instance. DNA methylation is one of reversible epigenetic modifications that responds to abiotic stresses and regulates the spatial and temporal expression of genes in the short-term and long-term. Understanding the mechanisms in depth could aid in the development of genetic tools to improve plant stress resistance and benefit us in molecular plant breeding. With the advance of current molecular technologies, our knowledge of plant epigenic responses to various stresses is growing rapidly. In this review, we discuss the recent advances in plant responses and adaptions to abiotic stresses, focusing on the DNA methylation response to stresses and its roles in plant stress memory.

The important physiological significance of cytosine DNA methylation in plant responses and adaptions to various abiotic stresses has been indicated in many studies. Changes in the DNA methylation state in specific regions or the global state regulate the expression of genes in response to stimuli requirements and improve their defense systems. However, more work on a wider range of plant species will be a benefit for us to gain a more complete understanding of the mechanisms of DNA methylation in plant evolution and adaption. With the advantages of the explosion data of genome-wide bisulfite DNA methylome data by next generation sequencing, a large-scale meta-analysis across plant species will provide us with more information about DNA methylation and plant abiotic stresses.

Plant stress memory and their capacity to influence plant tolerance to a changing environment and crop productivity have been considered to play an important role in the adaptation and evolution of plants [117]. However, there is still a knowledge gap in understanding the molecular mechanisms underlying the establishment and maintenance of plant stress memory, since such memories are not established in many cases, and how the ability maintained is not fully illustrated as well. Further studies on stress memory mechanisms will largely improve plant tolerance to rapidly changing environmental conditions.

The development of methodologies of epigenome editing tools that specifically target genomic loci to alter epigenetic modifications, such as cytosine methylation and demethylation, could enable the precise generation of artificial epigenomic diversity and site-specific epigenetic changes. Methods such as chemical treatment, target-specific epigenetic engineering, including exogenous RNAi mediated by virus-induced gene silencing and the CRISPR-Cas9 system, will also facilitate the efforts for epibreeding in the future [118,119,120].
